# From Pharmacological Treatment to Neuromodulation: A Comprehensive Approach to Managing Gilles de la Tourette Syndrome

**DOI:** 10.3390/ijms26188831

**Published:** 2025-09-10

**Authors:** Edoardo Monfrini, Christian Saleh, Domenico Servello, Phillip Jaszczuk, Mauro Porta

**Affiliations:** 1 Neurology Unit, Fondazione IRCCS Ca’ Granda Ospedale Maggiore Policlinico, 20122 Milan, Italy; edoardo.monfrini@hotmail.it; 2 University of Basel, 4001 Basel, Switzerland; 3 Centro Malattie Extrapiramidali e Sindrome di Tourette, IRCCS Ospedale Galeazzi-Sant’Ambrogio, 20122 Milan, Italy; servello@libero.it (D.S.); mauroportamilano@gmail.com (M.P.); 4 Interdisciplinary Spine Center, Luzern Cantonal Hospital, University of Luzern, 6000 Luzern, Switzerland; philja.ja@gmail.com

**Keywords:** Gilles de la Tourette syndrome, pharmacotherapy, deep brain stimulation, Habit Reversal Therapy

## Abstract

Gilles de la Tourette syndrome (GTS) is a neurodevelopmental disorder characterized by motor and phonic tics, often including attention deficit, hyperactivity, and obsessive–compulsive behaviours. The pathophysiology involves the dysfunction of cortico-striato-thalamo-cortical circuits, primarily implicating dopaminergic hyperactivity, but also involving multiple different neurotransmitter systems. Treatment of GTS is complex, highly individualized, and influenced by considerable variability in symptom presentation. Behavioural approaches, such as Habit Reversal Therapy (HRT), play a key role, especially in milder cases. Pharmacological therapy is largely empirical and varies across countries, influenced by drug availability and the perceived risks of certain classes of drugs, particularly dopamine receptor blocking agents. Drug options for managing tics include dopamine receptor antagonists, monoamine depleting agents, and alpha-2 agonists, all of which require close monitoring for metabolic, cardiovascular, and neurological side effects. Botulinum toxin injections represent an effective solution for focal tics that are resistant to systemic treatments. Cannabinoids and antiepileptics have limited efficacy, yet they may still offer relevant therapeutic potential in selected cases. Serotonergic drugs are useful for treating obsessive–compulsive symptoms. For patients with refractory tics, deep brain stimulation (DBS) represents an intervention of last-resort; however, DBS remains off-label and consensus on optimal targets is lacking. This narrative review draws on both the relevant literature and extensive personal clinical experience to explore the complexities of managing GTS, with a focus on evidence-based treatments for tics and associated neuropsychiatric symptoms. A therapeutic algorithm is proposed, emphasizing a “start low, go slow” approach, combining pharmacological interventions with cognitive behavioural and surgical therapies, when needed. We underscore the importance of tailoring treatments to individual patient profiles and symptom variability over time, highlighting the need for further research in GTS management.

## 1. Introduction

Gilles de la Tourette syndrome (GTS) is a neurodevelopmental disorder beginning in childhood or adolescence characterized by multiple motor tics and at least one phonic tic [1]. A tic is a repeated and largely involuntary movement or utterance varying in complexity from a muscle twitch or a grunt to elaborate well-coordinated actions and repeated words or phrases [2]. GTS often includes obsessive–compulsive behaviors (OCB) or obsessive–compulsive disorder (OCD), attention-deficit and hyperactivity disorder (ADHD), anxiety, mood disorders, oppositional behavior, aggression, and, in some cases, self-injurious behaviors (SIB) [1,3].

The core neuroanatomical substrate implicated in GTS is the cortico-striato-thalamo-cortical (CSTC) circuitry, which regulates motor control, inhibition, and habit formation [4]. Within this framework, dopaminergic signaling in the striatum is thought to modulate the balance between the direct and indirect pathways, thereby influencing tic initiation and suppression [5]. Dopaminergic hyperactivity has long been considered a central feature of GTS pathophysiology. Positron emission tomography (PET) and single-photon emission computed tomography (SPECT) studies in individuals with GTS have shown increased striatal D2 receptor binding, particularly in the putamen, consistent with postsynaptic dopaminergic receptor upregulation. Findings from animal models further support this notion, suggesting an imbalance between the activity of D1- and D2-mediated pathways within the striatum, which may disrupt the normal inhibition of competing motor programs and contribute to tic expression [5]. Glutamatergic projections from the cortex to the striatum provide excitatory input, while GABAergic interneurons and output pathways mediate inhibitory control [6]. Serotonergic modulation, primarily from the raphe nuclei, influences both cortical and subcortical excitability, with downstream effects on motor and emotional regulation [7]. Several studies highlight the potential role of the histaminergic system in tic pathophysiology, particularly through alterations in histidine decarboxylase (HDC), the rate-limiting enzyme in histamine biosynthesis [8]. Rare pathogenic variants in *HDC* have been identified in individuals with GTS, and Hdc-knockout mice exhibit tic-like behaviors and deficits in sensorimotor gating, suggesting that impaired histamine signaling may disrupt the striatal and cortical circuits involved in motor control [8,9]. More broadly, genetic studies have implicated other rare variants associated with GTS, including *SLITRK1*, *NRXN1*, and *CNTN6*, which point to abnormalities in synaptic development and cortico-striatal connectivity [10]. Disruption in the coordination of these systems—rather than dysfunction in any single neurotransmitter—is increasingly recognized as a key factor in the pathophysiology of tic disorders [4,11]. Taken together, these findings support the hypothesis that GTS may result from a convergence of neurodevelopmental and neurochemical alterations, with variability in the underlying mechanisms across individuals. This biological heterogeneity may have implications for the development of more targeted and individualized therapeutic approaches [12].

Given the heterogeneous and fluctuating nature of symptoms due to the interplay of multiple neurobiological systems, the neuropsychopharmacological treatment of GTS remains a challenge. This narrative review focuses on the complex management of GTS, including the treatment of tics and coexisting clinical manifestations, such as OCB and attention deficit hyperactivity disorder (ADHD) [13], aiming to provide a clinically oriented synthesis of current pharmacological and neuromodulatory treatment strategies for GTS. This approach combines a critical analysis of the available literature with insights derived from extensive clinical experience. This format is intended to offer practical guidance for clinicians, particularly in an area where high-level evidence is lacking.

## 2. A Glimpse into the Complexity of GTS

The therapeutic approach to GTS is inherently complex and necessitates the consideration of multiple factors.

First, pharmacological treatment for GTS remains largely empirical, relying primarily on case series and open-label trials with few exceptions. Most medications used to treat tics do not have specific indications for GTS and are often restricted to psychiatric use [13]. This poses significant challenges as general practitioners may refuse to prescribe off-label medications, complicating access to treatment. Moreover, many of these drugs are not reimbursed by medical insurances and national healthcare systems, making affordability another major barrier [14,15].

Second, tics often wax and wane over time. Consequently, treatment should be dynamic and tailored to the patient’s needs, incorporating periods of observation where medication is withheld, followed by the introduction of pharmacotherapy when symptoms become disabling [16]. Given this variability, tracking the clinical course of GTS symptoms over time using a longitudinal plot can provide a valuable visual representation of fluctuations, treatment responses, and periods of exacerbation or remission [17].

Third, clinicians should recognize when pharmacological treatment should be complemented by behavioral interventions such as Habit Reversal Therapy (HRT), which can also serve as an effective standalone treatment, especially in patients who are consciously aware of their premonitory urges and exhibit a limited tic repertoire [18].

Fourth, while tics are the pathognomonic symptoms of GTS, treatment strategies must also address other symptomatic domains, such as attention deficit/hyperactivity disorders, impulsivity, obsessions, compulsions, aggression and SIB. Identifying the condition that contributes most to impairment is essential, and, in many cases, a combination of therapies is necessary to effectively manage GTS [13]. Notably, the nosographic classification of ADHD and OCD in the context of GTS remains complex and debated, as some authors consider them distinct comorbidities, while others view them as intrinsic manifestations of the syndrome itself, hypothesizing shared genetic and neurobiological underpinnings and challenging the traditional boundaries between primary and associated disorders [19]. In this regard, Dell’Osso and colleagues proposed Obsessive–Compulsive Tic Disorder (OCTD) as a particularly complex neuropsychiatric syndrome lying at the intersection of GTS and OCD [20].

Fifth, compliance remains a significant challenge in GTS management, affecting treatment efficacy and patient outcomes. Poor adherence can stem from multiple sources, including patients not taking medications as prescribed, mainly due to concerns about side effects, such as weight gain and sedation, or caregivers being overly cautious about administering drugs with “intimidating” names. The therapeutic alliance between physician, patient, and family is critical to improving adherence, particularly in pediatric cases [21].

Sixth, while many patients respond well to first-line agents, a subset remains resistant to pharmacological interventions (refractory GTS). At our centers, refractoriness is defined by failing to respond to HRT and at least three drugs of the following classes: typical and/or atypical antipsychotics, monoamine depletors, and selective serotonin reuptake inhibitors (SSRI). In some cases, they may develop “status ticcosus,” a rare but severe condition characterized by continuous, intense, and disabling tics. In such cases, experimental treatments, immunomodulatory approaches, or advanced neuromodulation techniques, such as deep brain stimulation (DBS), may be warranted [13,22].

Seventh, the “referred Tourette” phenomenon, where patients report frequent and severe tics at home but do not display any tics during the clinical evaluation, complicates further therapeutic decisions. Prescribing medication without directly observing the symptoms raises ethical concerns about over- or under-treatment.

Ultimately, neuropsychopharmacology in GTS remains an evolving field and fundamentally relies on a multidisciplinary approach. Effective management requires the combined expertise of pediatric neuropsychiatrists, neurologists, psychiatrists, behavioral therapists, and neurosurgeons, each playing a crucial role in tailoring treatment to the patient’s specific needs. From a clinical standpoint, one of the most challenging aspects of GTS is the striking interindividual variability in symptom presentation, disease trajectory, and treatment response. In our experience, this heterogeneity often defies rigid treatment algorithms and demands highly personalized strategies based on long-term observation and iterative adjustment. Given the heterogeneity of symptom presentation and treatment response, a coordinated, multimodal approach is essential, ensuring that interventions are holistic, adaptable, patient-centered, and based on close monitoring (i.e., regular follow-up visits) [23].

## 3. General Approach to Tics in GTS

Therapeutic intervention in GTS is warranted when tics significantly impact social interactions, academic or work performance, and daily activities, or lead to discomfort, pain, or injury [24]. The primary objective of treatment is to decrease tic frequency, enhancing overall function and quality of life with a particular emphasis on reducing social impairment. As a result, therapeutic success cannot be defined in universal terms but must instead be individually tailored to each patient [21].

**Figure 1:**
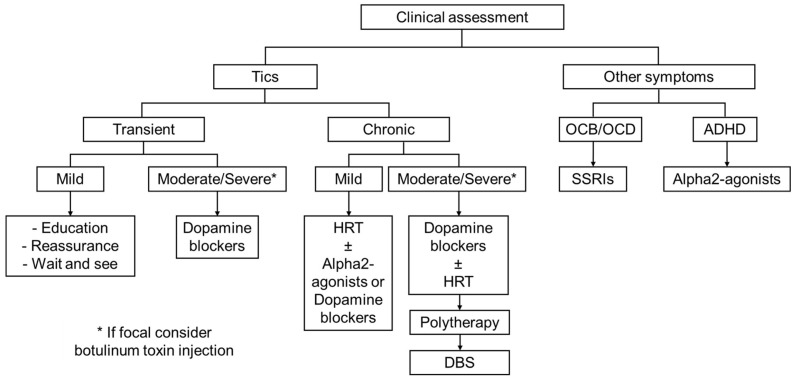
Practical algorithm for GTS management. However, it is important to recognize that a purely symptom-based approach is often inadequate. Effective management of GTS must take into account not only symptom severity, but also psychosocial functioning, family dynamics, and the individual priorities of the patient. In this context, clinical judgment and flexibility frequently prove more valuable than rigid adherence to stepwise treatment protocols.

In our experience, therapeutic approach should follow a stepwise strategy based on the severity of tics (i.e., impact on daily life) (Figure 1). If tics are transient, mild and non-disabling, a watchful waiting approach should be recommended, as they may resolve spontaneously. Patients should receive education, counselling, and supportive care. In cases of chronic tics (>1 year), management depends on their severity. Mild tics often require only behavioral interventions such as HRT or simple reassurance. For moderate to severe tics, treatment options include behavioral therapy (i.e., HRT), pharmacological therapy, or a combination of both. If monotherapy is insufficient, polytherapy may be necessary, with careful consideration of potential pharmacological interactions.

The principle of “start low, go slow” is crucial, beginning with low doses and titrating upward cautiously to minimize side effects. Before initiating treatment, appropriate screening tests, such as ECG, weight monitoring, and laboratory analyses, should be performed [13].

Emerging evidence suggests that sex differences may influence tic severity and pharmacological responses, potentially due to the hormonal modulation of dopaminergic transmission, particularly during puberty. For example, androgens may enhance dopaminergic activity, contributing to the higher prevalence and severity of tics in males, while females may show different sensitivity to dopaminergic blockade, which could influence treatment tolerability and efficacy [25,26].

The duration of treatment should be tailored to each patient based on symptom severity, functional impact, comorbidities, and treatment response. As a rule, a gradual tapering to prevent withdrawal effects or symptom rebound should be considered if stability is maintained for several weeks/months [21]. Regular reassessment is crucial to determine the need for treatment adjustments, and a flexible, patient-centered approach—supported by the possibility of direct contact with the treating physician—ensures optimal symptom control while minimizing medication burden and side effects. Most patients experience spontaneous improvement in late adolescence or early adulthood, which often permits dose reduction or discontinuation [3]. In chronic disabling cases, long-term therapy may be necessary, especially in patients with OCD or ADHD. When these neuropsychiatric manifestations are present, targeted pharmacological interventions may be necessary, but they should be carefully chosen to minimize the risk of exacerbating tics [21]. In severe and refractory cases, neuromodulation (i.e., deep brain stimulation, DBS) may be a last-resort option, which we will discuss in this review.

## 4. Habit Reversal Therapy (HRT)

HRT was first described in 1973 by Azrin and Nunn in their seminal publication, “Habit Reversal: A Method of Eliminating Nervous Habits and Tics” [27]. HRT is a structured behavioral intervention designed to enhance the patient’s awareness of tics and to replace them with socially acceptable competing responses. It is typically delivered in an outpatient setting over a period three to four months, during which tic frequency is systematically monitored and recorded by both patients and caregivers to provide feedback and track progress [18].

The intervention consists of several core components, including awareness training with self-monitoring, relaxation techniques, and competing response training. A comprehensive review by Dutta and Cavanna (2013) concluded that HRT is an effective first-line behavioral therapy for GTS across all age groups [28]. Despite its demonstrated efficacy, the widespread implementation of HRT remains limited, in part due to the scarcity of clinicians adequately trained in its application [29].

Beyond its behavioral efficacy, HRT has been increasingly associated with measurable changes in brain function. Neuroimaging findings suggest that behavioral interventions such HRT may enhance activity and functional connectivity in premotor and sensorimotor regions, possibly reflecting neuroplastic changes that facilitate awareness of premonitory urges and voluntary suppression of tics [30]. These findings support the view that behavioral interventions such as HRT engage the same cortical–striatal circuits implicated in tic generation, offering a non-pharmacological avenue for rebalancing dysfunctional motor pathways.

## 5. Pharmacology of Tics

### 5.1. Dopamine Receptor Antagonists

Dopamine receptor blockers, also known as antipsychotics and previously known as neuroleptics, exert their therapeutic effect by antagonizing dopamine D2 receptors in the basal ganglia [31]. They have long been the cornerstone of GTS treatment. Among them, haloperidol, pimozide, and aripiprazole are the only agents approved by the FDA for GTS, while, in Europe, only haloperidol has received regulatory approval [32]. Although highly effective in reducing tics, their use is often constrained by significant short- and long-term adverse effects, necessitating careful patient selection and monitoring [24].

First-generation antipsychotics, such as haloperidol, pimozide and fluphenazine, are potent D2 receptor antagonists that significantly reduce tic severity but are associated with a high risk of extrapyramidal side effects (EPS), including parkinsonism, dystonia, akathisia, and tardive dyskinesia [24]. Haloperidol has largely fallen out of favor due to its substantial adverse effect profile, including electrocardiographic changes (ECG), foremost QTc prolongation and the related increased risk of dysrhythmia (Torsades de Pointes), apathy, anhedonia, and metabolic disturbances [33]. Pimozide, while somewhat better tolerated than haloperidol, also carries a risk of QTc prolongation, sedation, and weight gain [34]. Fluphenazine has shown moderate efficacy with a lower risk of tardive dyskinesia, although it remains associated with drowsiness and weight gain [35].

Second- and third-generation antipsychotics are now preferred due to their broader receptor profile, including antagonism of serotonin receptors (5HT2A and 5HT2C), which may contribute to a more favorable tolerability profile [36]. However, these agents pose an increased risk of metabolic disturbances such as weight gain, dyslipidemia, hyperglycemia, and diabetes [37]. Weight gain may significantly impact treatment adherence and overall quality of life. The underlying mechanism of antipsychotic-induced weight gain is believed to involve antagonism of hypothalamic histamine H1 and serotonin 5-HT2C receptors, which disrupt appetite regulation and energy balance by increasing food intake and reducing satiety [38]. Additional contributions from dopaminergic and orexinergic pathways may further modulate this metabolic dysregulation [39]. Risperidone, the most well-studied second-generation antipsychotic for GTS, demonstrates strong efficacy but requires monitoring for EPS and metabolic side effects, including hyperprolactinemia [40]. Aripiprazole, a third-generation antipsychotic, has gained prominence as a preferred option due to its favorable balance of efficacy and tolerability, although it remains associated with weight gain and metabolic alterations [41].

The side effects of antipsychotics are particularly relevant and need to be particularly carefully considered, given that individuals with GTS may already have an elevated predisposition to metabolic and cardiovascular comorbidities. Therefore, a careful risk–benefit assessment is crucial, particularly in pediatric populations, where long-term adverse effects may have lasting consequences [42].

Dopamine receptor antagonists present several pharmacological interactions. Their combination with central nervous system (CNS) depressants such as benzodiazepines can lead to increased sedation and a higher risk of respiratory depression [43]. Selective serotonin reuptake inhibitors (SSRIs) may elevate plasma levels of pimozide and risperidone by inhibiting CYP2D6, increasing the risk of QTc prolongation and EPS [44]. Concurrent use with QTc-prolonging drugs, including macrolide antibiotics, antiarrhythmics, tricyclic antidepressants, or ondansetron, further raises the risk of torsades de pointes [43].

Despite the widespread use of antipsychotics in GTS, there remains an ongoing search for alternative dopaminergic agents with improved safety profiles. Experimental therapies, such as ecopipam, a selective D1 receptor antagonist, have shown promise in reducing tic severity in early trials. However, further research is needed to determine its efficacy and safety in routine clinical practice [45,46].

### 5.2. Monoamine Depletors

Monoamine depletors act by inhibiting the vesicular monoamine transporter 2 (VMAT2), leading to the presynaptic depletion of dopamine and other monoamines. They are increasingly used in the treatment of GTS due to their effectiveness in reducing tics and their lower risk of tardive dyskinesia compared to dopamine-receptor-blocking agents [47].

Tetrabenazine, an FDA-approved treatment for chorea in Huntington’s disease, has shown promise in open-label trials for tic management [48]. However, its use is limited by side effects such as drowsiness, parkinsonism, akathisia, depression, and a potential risk of suicidality [49]. Deutetrabenazine, a deuterated form of tetrabenazine, offers pharmacokinetic advantages, including less frequent dosing and improved tolerability [50]. Pilot studies have demonstrated that deutetrabenazine significantly reduces tic severity and enhances quality of life in adolescents with GTS with a more favourable safety profile [51]. Valbenazine has also been investigated for GTS, but clinical trials have yielded inconsistent results, possibly due to factors such as mild case inclusion or suboptimal dosing [52].

When combined with antipsychotics, their additive dopamine-depleting effects may heighten the risk of parkinsonism, dystonia, and akathisia [43]. Co-administration with SSRIs such as fluoxetine, paroxetine, or sertraline can inhibit their metabolism, leading to increased systemic levels and exacerbating side effects including sedation, depression, and suicidal ideation [43,44]. Additionally, their interaction with beta-blockers such as propranolol and metoprolol may further increase the risk of bradycardia and hypotension [53,54].

Although randomized, double-blind, placebo-controlled trials for VMAT2 inhibitors in GTS are lacking, many clinicians consider them a first-line option for patients with troublesome tics. Careful monitoring and individualized dosing strategies are crucial, particularly in pediatric populations. These agents provide an important alternative for managing tics, especially in patients who do not tolerate or respond well to dopamine receptor antagonists [47].

### 5.3. Alpha-2 Receptor Agonists

Alpha-2 agonists activate presynaptic alpha-2 adrenergic receptors, reducing norepinephrine release and sympathetic outflow [55]. Agents such as clonidine and guanfacine, initially developed to treat arterial hypertension, are often utilized to manage patients with newly diagnosed GTS [56]. Guanfacine exhibits greater selectivity for α2A-adrenergic receptors, which are predominantly expressed in the prefrontal cortex, contributing to its efficacy in enhancing attention and impulse control with fewer sedative and hypotensive side effects. In contrast, clonidine activates α2A, α2B, and α2C receptors, the broader activity of which may underlie its greater sedation and risk of blood pressure reduction [57]. These medications demonstrated modest efficacy in reducing tics, with a stronger clinical benefit in cases where tics co-occur with attention deficit, hyperactivity, impulse control disorder and rage attacks [58]. Clonidine can also be particularly helpful for facilitating sleep onset, though daytime sedation is a limiting factor [59]. When combined with CNS stimulants like methylphenidate, alpha-2 agonists may offer enhanced control of both tics and ADHD without worsening tics [60].

Clonidine, available in oral and transdermal forms, is effective but associated with more frequent sedation, fatigue, and other side effects. Guanfacine is often preferred due to its lower sedative effect and similar efficacy in managing coexisting ADHD and impulse control issues. However, the effectiveness of these medications in treating tics is limited when ADHD or other comorbidities are absent [56].

Side effects of this class commonly include sedation, fatigue, dry mouth, headaches, and irritability, with rare occurrences of bradycardia and orthostatic hypotension. Abrupt discontinuation may trigger rebound hypertension, which is thought to result from sudden loss of presynaptic inhibition of norepinephrine release, leading to sympathetic overactivity and elevated vascular tone. When combined with CNS depressants, including benzodiazepines, antipsychotics, and sedating antidepressants, they can intensify sedation and lead to hypotension. Additionally, co-administration with antihypertensive agents, such as beta-blockers or calcium channel blockers, increases the risk of excessive hypotension and dizziness, necessitating cautious dose adjustments and monitoring [55].

Despite these limitations, alpha-2 agonists may be a reasonable option for patients with mild tics that are troublesome enough to require pharmacologic intervention. For GTS with ADHD, combining alpha-2 agonists with CNS stimulants may yield optimal outcomes [58].

### 5.4. Botulinum Toxin

Botulinum toxin acts by inhibiting acetylcholine release at the neuromuscular junction, leading to temporary chemodenervation and muscle relaxation at the site of injection [61]. Botulinum toxin injections (BTI) have been utilized for years to address focal motor tics and certain phonic tics, including complex ones such as coprolalia [62]. Although rigorous studies are limited, open-label trials and case series have consistently demonstrated their benefits. These include reducing tic frequency and severity, alleviating regional premonitory urges, and improving quality of life for many patients [63].

Clinical observations suggest that botulinum toxin is particularly effective for localized tics that do not respond well to oral medications. For example, it has shown success in managing repetitive cervical extension tics (“whiplash tics”), which can lead to complications including compressive cervical myelopathy or vertebral artery dissection [64].

The effects of botulinum toxin are temporary, typically lasting several months, with most patients experiencing significant relief. Adverse effects, such as focal muscle weakness or hypophonia, are generally mild and transient. Despite these side effects, botulinum toxin remains a valuable option for managing refractory focal tics. A time interval of at least three months between BTI doses needs to be respected to avoid resistance [65].

### 5.5. Antiseizure Drugs

Antiseizure drugs modulate neuronal excitability via diverse mechanisms, including enhancement of GABAergic inhibition and modulation of ion channels [66]. This class of drugs have been explored as a potential treatment for GTS, but none have become standard therapy [67]. Among these, topiramate has demonstrated promising effects in reducing tic severity and improving premonitory urges. However, its use is associated with side effects such as drowsiness, appetite and weight loss and cognitive dysfunction, which can limit its applicability, particularly in children [68]. Levetiracetam has also been studied, showing potential in initial trials, but its efficacy has not been consistently supported by more rigorous randomized controlled studies [69,70]. In highly selected cases, antiepileptic polytherapy may be considered, mirroring strategies employed in other neurological conditions such as trigeminal neuralgia, where the combination of multiple agents can enhance therapeutic efficacy [71]. While these drugs may offer benefits for some patients, the current evidence is insufficient to recommend them as routine treatment for GTS. Further high-quality research is needed to clarify their role in managing this condition.

### 5.6. Cannabinoids

Cannabinoids exert their effects by modulating the endocannabinoid system, primarily through CB1 receptor activation in the central nervous system, influencing dopaminergic and GABAergic transmission [72]. Cannabis and cannabinoids as potential treatments for GTS have gained momentum in recent years. Patients frequently report subjective improvements in tics with cannabis use, but these responses are often nonspecific and challenging to validate scientifically [13].

Current findings suggest only limited effectiveness of pharmaceutical cannabidiol (CBD), delta-9-tetrahydrocannabinol (THC) or medicinal cannabis in the management of tics [73]. Preliminary results from experimental treatments, such as SCI-110 (a combination of dronabinol and palmitoylethanolamide), have shown promise, meeting primary endpoints in early studies. Another agent, ABX-1431, which inhibits monoacylglycerol lipase to modulate endogenous cannabinoid activity, has demonstrated modest tic reductions in small trials [74]. Further research into these novel therapies is ongoing.

Despite growing interest, cannabinoids are not yet established as a reliable or routine treatment for GTS. Comprehensive high-quality research is needed to confirm their therapeutic potential and clarify their role in managing GTS symptoms [13]. Furthermore, cannabis can induce significant psychological adverse effects, i.e., psychosis [75].

### 5.7. GABAergic Drugs

Clonazepam, a benzodiazepine that enhances GABA-A receptor activity through positive allosteric modulation, has been explored as a treatment for GTS [56]. In addition to its GABAergic effects, clonazepam also seems to influence the serotonergic system [76]. Some open-label studies and a small comparative trial suggest potential benefits in tic suppression, but its widespread use is limited by the absence of placebo-controlled trials, the risk of tolerance, and side effects such as sedation, memory impairment, and paradoxical disinhibition [77,78].

Baclofen, a GABA-B receptor agonist primarily used for spasticity, has shown promising results in an open-label study, with most children experiencing tic reduction [79]. However, a small, randomized trial failed to demonstrate significant improvements in tic frequency or severity. Sedation and drowsiness remain common side effects [80]. Overall, while GABAergic agents are not first-line treatments for GTS, they may have a role in carefully selected cases.

### 5.8. Nicotine

Nicotine acts as an agonist at nicotinic acetylcholine receptors, influencing dopaminergic and attentional pathways [81]. Case reports indicate tic worsening after smoking cessation, while trials with nicotine gum and transdermal patches have shown reductions in tic frequency and severity, along with improvements in attention. However, benefits were often short-lived, and side effects including nausea, headache, and sedation limited tolerability [82,83,84,85]. Despite its potential, nicotine is not a standard treatment due to inconsistent efficacy and side effects.

## 6. Pharmacology of Non-Motor Symptoms

### 6.1. Obsessive–Compulsive Spectrum

For GTS patients with OCB, cognitive-behavioral therapy (CBT) is the first-line treatment, either alone or in combination with selective serotonin reuptake inhibitors (SSRIs) [86]. Commonly used SSRIs include sertraline, fluoxetine, and fluvoxamine [86]. While these medications do not seem to directly target tics, they alleviate obsessive–compulsive symptoms and anxiety, which can indirectly reduce the severity of tic-related symptoms [86]. Although SSRIs are commonly used to treat anxiety and obsessive–compulsive symptoms in GTS, they have occasionally been reported to exacerbate tics in a subset of patients [87]. This paradoxical effect may be explained by the functional interplay between serotonergic and dopaminergic systems, particularly within prefrontal–striatal circuits. SSRIs increase extracellular serotonin, which can indirectly modulate dopaminergic tone. In vulnerable individuals, enhanced serotonergic input to the prefrontal cortex may lead to reduced inhibitory control over subcortical dopaminergic pathways, potentially resulting in the disinhibition of tic-related motor programs [7]. While not universally observed, this mechanism highlights the importance of individualized pharmacological strategies and close monitoring when prescribing serotonergic agents in GTS.

Small studies with serotonergic antagonists, such as ketanserin and ondansetron, have also reported some benefits [88,89]. Pimavanserin, a relatively novel serotonin inverse agonist, selectively targets 5-HT2A receptors, offering a potential therapeutic avenue without directly affecting dopamine. Initially approved for Parkinson’s-associated psychosis, pimavanserin is now being investigated for GTS, providing a promising alternative for modulating serotonergic activity [90]. Antipsychotics may be an option for persistent severe OCB/OCD despite these interventions [56]. Importantly, Aripiprazole, a partial dopamine D2 agonist, can be beneficial for OCB when used alongside SSRIs but it may also worsen compulsive behaviors, possibly due to its dopaminergic effects in brain regions involved in OCD. This paradoxical effect is more likely when aripiprazole is used as monotherapy at lower doses, potentially increasing dopamine signaling in the mesolimbic system [91]. In rare cases of severe, treatment-resistant OCD, DBS may be considered [92].

### 6.2. Attention Deficit and Hyperactivity Spectrum

In preschool children (four to five years) with GTS complicated by ADHD, behavioral interventions are preferred as the first-line approach, while, in school-aged children and adolescents, they are often used alongside medication [92]. When pharmacologic treatment is needed, alpha-adrenergic agonists such as guanfacine and clonidine are favored as they address both tics and ADHD symptoms [58]. If central nervous system stimulants (e.g., methylphenidate) are necessary, many clinicians prefer to first stabilize tics with an alpha-adrenergic agonist before introducing stimulants, as these drugs can sometimes exacerbate tics [58]. Atomoxetine, a non-stimulant, is considered only modestly effective for ADHD in GTS [93].

## 7. The Case for Neuromodulation in GTS

When pharmacological interventions fail to provide adequate symptom control in GTS, DBS may be considered as a last-resort therapeutic option [94]. Despite over four decades of clinical use of DBS for motor and psychiatric disorders, its mechanisms of action remain incompletely understood. Initially hypothesized to exert a purely inhibitory effect—drawing parallels with ablative surgical procedures—subsequent evidence has revealed a more complex neuromodulatory role, involving both inhibitory and excitatory influences on neural circuitry [95]. Several mechanistic hypotheses have been proposed to explain the clinical efficacy of DBS in GTS. One suggests that DBS may induce long-term neuroplastic changes, consistent with the observation that symptomatic improvement typically emerges gradually over time [96]. Another posits that, rather than simply altering neuronal firing rates, DBS may serve to restore the synchrony or coherence of pathological network activity [95]. Additionally, functional imaging studies, including [^18^F] fallypride positron emission tomography (PET), have demonstrated a reduction in striatal dopamine release following DBS [97]. While basic research continues to explore the neurobiological underpinnings of GTS, further investigations are warranted to delineate the precise therapeutic mechanisms of DBS. In the meantime, clinical observation remains the principal guide for DBS use in this population.

The modern application of DBS in GTS was pioneered in 1999 by Vandewalle et al., who targeted the thalamus in a single patient [98]. Since this landmark case, approximately 350 individuals worldwide have undergone DBS for GTS, with many reporting reductions exceeding 50% in Yale Global Tic Severity Scale (YGTSS) scores [99]. Despite these encouraging outcomes, DBS for GTS remains an off-label intervention, lacking both Conformité Européenne (CE) marking and U.S. Food and Drug Administration (FDA) approval.

**Figure 2:**
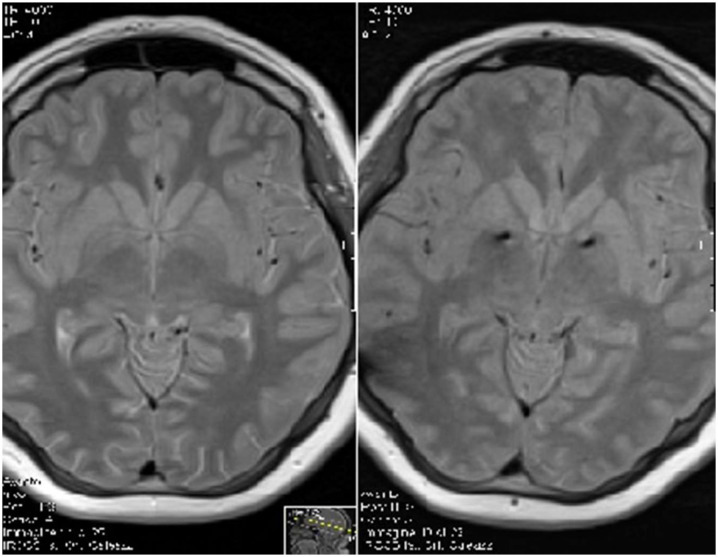
Axial T2-weighted MRI sequences before (**left**) and after surgery (**right**), showing electrodes in the anteromedial part of globus pallidus internus (bilateral hypointense signal). The images were obtained from anonymized MRI scans of one of our patients treated with DBS as part of our institutional case series. The image was selected for illustrative purposes only and does not allow for patient identification [103].

To date, no consensus has been reached regarding the optimal anatomical target for stimulation. Based on clinical experience and accumulated data, the medial (limbic) aspect and the anterior part of the globus pallidus internus (GPi) appear to be the most promising targets (Figure 2). Alternative targets, applied either singly or in combination, have included the posteroventral (motor) GPi, thalamus, the anterior limb of the internal capsule (ALIC), nucleus accumbens (NA), and the subthalamic nucleus (STN), with varying degrees of clinical success [99]. The Montpellier groupintroduced a dual-targeting approach involving the simultaneous implantation of electrodes in both the medial and dorsolateral GPi, aimed at addressing the limbic and motor symptomatology of GTS, respectively, however, only one single case was reported [100,101]. At our institution, we have developed the concept of “rescue surgery,” referring to a second surgical intervention performed in refractory patients who derive insufficient benefit from their initial DBS implantation [102]. One of the major challenges in identifying a universal target is the significant inter-individual variability in the neuropsychiatric comorbidities associated with GTS, including OCB, OCD, ADHD, depression, and anxiety [102]. Whether these comorbidities should serve as exclusion criteria for DBS remains a matter of ongoing debate.

The appropriate age for surgical intervention is also contentious, with the typical range of implantation occurring between 18 and 25 years [104]. However, we argue against a rigid age threshold and advocate for early intervention in carefully selected severe refractory cases, especially considering that GTS often peaks in severity between 11 and 14 years—a critical period for neurodevelopment and social integration. Within this context, we have proposed the concept of “bridging therapy”, whereby DBS is implanted at the peak of tic severity and electively explanted once symptoms have subsided following close clinical monitoring [105]. This strategy may offer significant benefits in terms of avoiding the long-term psychosocial consequences of undertreated GTS, as “wait-and-see” approaches may inadvertently lead to lasting impairment in quality of life and social functioning. Indeed, DBS not only reduces tic severity but can also restore social engagement, with patients resuming academic, occupational, and interpersonal activities [106].

A significant limitation in the current DBS literature for GTS is the presence of publication bias, disproportionately emphasizing positive outcomes, thereby limiting the generalizability and objectivity of the evidence base [104]. To ensure the reliability of future meta-analyses and clinical guidelines, it is essential that both therapeutic failures and surgical complications are systematically and transparently reported. As with all interventional procedures, DBS carries inherent surgical risks, most of which are related to stimulation and typically transient in nature [99]. A rigorous and balanced risk–benefit assessment must be undertaken and clearly communicated to both patients and caregivers prior to surgical decision making. Ultimately, the success of DBS in GTS depends not only on the procedure itself but also on the adoption of a comprehensive multidisciplinary approach, involving close collaboration among a neurologist, a clinical psychologist with expertise in GTS, and an experienced functional neurosurgeon [96].

In addition to DBS, several other neuromodulatory strategies have been explored in the management of GTS, particularly in patients with refractory symptoms. Among these, vagus nerve stimulation (VNS) has been tested in isolated cases, with early reports suggesting potential benefits in tic reduction, likely through the modulation of thalamocortical and limbic circuits. Notably, Diamond et al. described clinical improvement in a GTS patient treated with VNS [107]. More recently, VNS has been explored in small series or as part of broader neuromodulation trials, though no large-scale or controlled studies have confirmed its efficacy.

Other non-invasive neuromodulation techniques are also under investigation. Repetitive transcranial magnetic stimulation (rTMS), particularly when applied to the supplementary motor area (SMA), has shown modest and variable effects on tic severity in small, controlled studies, but the results are heterogeneous and not yet conclusive [108]. Similarly, transcranial direct current stimulation (tDCS) has been explored as a means of modulating cortical excitability in GTS, with preliminary data suggesting it may reduce tics and improve premonitory urges in some individuals [109]. However, methodological inconsistencies and small sample sizes limit the generalizability of findings for both rTMS and tDCS. Taken together, these emerging neuromodulation strategies offer promising avenues for future research, but they currently lack sufficient evidence to support routine clinical use. They may be considered in highly selected or research settings, particularly where standard pharmacological options are inadequate or not tolerated.

## 8. Conclusions

**Figure 3:**
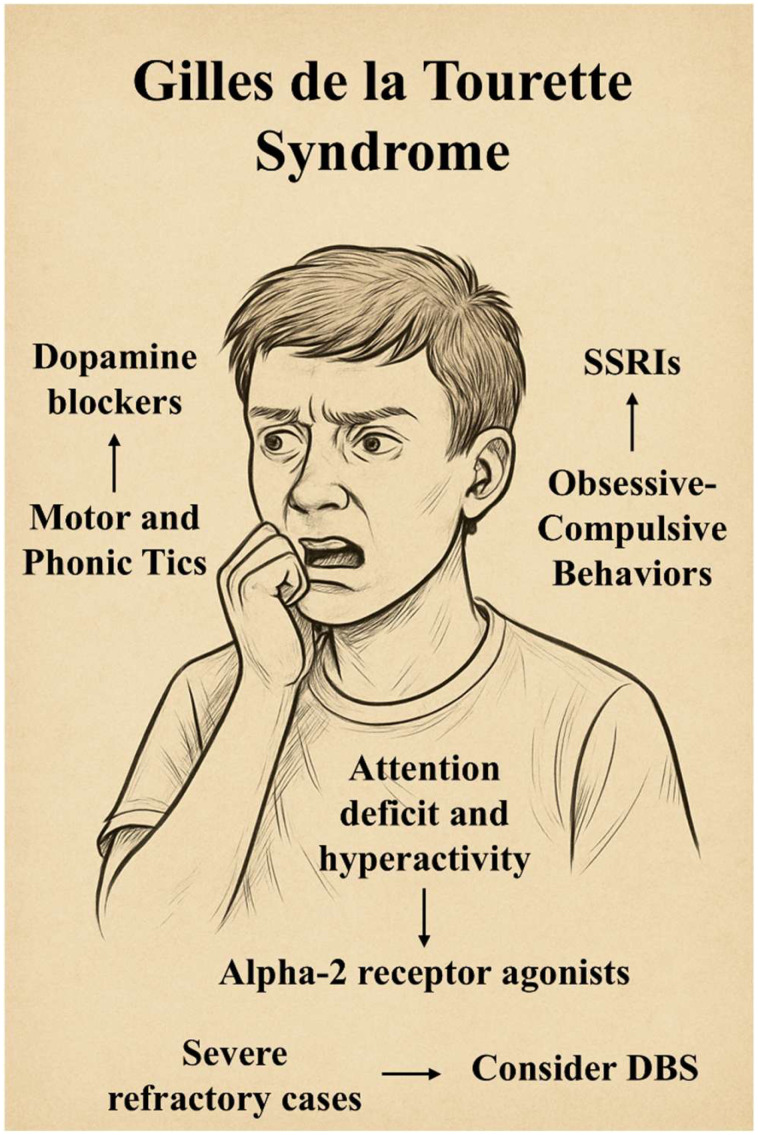
Overview of key symptom domains in Tourette syndrome and corresponding treatment strategies.

In summary, the management of GTS remains an intricate and evolving challenge, necessitating a nuanced and individualized approach (Figure 3). The complexity of GTS arises from its heterogeneous symptomatology, fluctuating course, and relevant non-motor symptoms, requiring clinicians to balance tic suppression with the broader neuropsychiatric needs of each patient. Importantly, the definition of treatment success in GTS should not rely solely on clinical scales but must encompass the broader psychosocial impact on patient functioning and well-being. Ultimately, the most effective treatment strategy is one that is patient-centered, multidisciplinary, and adaptable, integrating pharmacological, behavioral, and surgical approaches where necessary. Continued research and collaboration will be key to transforming our understanding of this complex disorder and enhancing the quality of life for those affected.
